# The Evaluation of the Effect of ICT in HIV Prevention in the General Population in China Based on an Information-Motivation-Behavioral Skill Model

**DOI:** 10.1155/2020/8786467

**Published:** 2020-10-29

**Authors:** Xia Liang, Jun Yang, Abu S. Abdullah, Zhikui He, Li Yang

**Affiliations:** ^1^School of Public Health, Guangxi Medical University, Nanning, Guangxi Province 530021, China; ^2^The Second People's Hospital of Nanning City, The Third Affiliated Hospital of Guangxi Medical University, Guangxi, Nanning, Guangxi Province 530022, China; ^3^Boston University School of Medicine, Boston Medical Center, Boston, Massachusetts 02118, USA; ^4^Duke Global Health Institute, Duke University, Durham, North Carolina 27710, USA; ^5^Global Health Program, Duke Kunshan University, Kunshan, Jiangsu Province 215347, China

## Abstract

**Objectives:**

With an increase in human immunodeficiency virus (HIV) infection, the application of information and communication technology (ICT) is considered as a helpful solution. The aim of this study is to evaluate the effect of ICT in HIV prevention in the general population based on an information-motivation-behavioral skill (IMB) model.

**Methods:**

A national follow-up study on the evaluation of ICT on HIV health education effects before and after large sample intervention was conducted in 16 provinces of China. ICT was used to carry out a six-month educational program on the prevention of HIV for participants using the WeChat platform. The research group conducted a second questionnaire for participants to collect data and built the IMB model using Mplus 7.0 analysis software.

**Results:**

A total of 997 questionnaires were sent, and 957 effective questionnaires were recovered, a recovery rate of 96.0%. Before the ICT intervention, the scores of information was 15.68 ± 3.28, of motivation was 14.47 ± 4.26, of behavior skills was 2.26 ± 1.08, and of condom use was 2.19 ± 1.15. After the ICT intervention, the scores for information (18.54 ± 2.48), motivation (16.06 ± 4.11), behavior skills (4.74 ± 1.04), and condom use (2.64 ± 1.15) improved significantly. ICT use had a significant regression effect on motivation (*β* = 0.237, *P* < 0.001), behavior skills (*β* = 0.997, *P* < 0.001), and information (*β* = 0.441, *P* < 0.001), while motivation (*β* = 0.196, *P* < 0.001), behavior skills (*β* = 0.207, *P* < 0.001), and information (*β* = 0.092, *P* < 0.001) had a significant regression effect on condom use.

**Conclusions:**

The ICT intervention can not only improve information about HIV prevention, motivation, and behavior skills but also promote the use of condoms, so as to achieve the result of promoting behaviors that act to prevent AIDS within the general population.

## 1. Introduction

Information and communication technology (ICT) is a set of information technologies that enables users to process, store, and transmit information and can facilitate the process of information-related activities [1]. In recent decades, the application of ICT tools in the research and training of health sciences has grown rapidly worldwide in both developed countries and developing countries [2, 3], and ICT has been adopted in fields of healthcare and health research, proving beneficial to the public and business sectors, as well as the medical profession [4].

At present, human immunodeficiency virus (HIV) infection and acquired immune deficiency syndrome (AIDS) are still major public health concerns. As of September 30, 2018, there were 849,602 HIV/AIDS patients and 262,442 HIV-related deaths reported in China, along with 497,231 HIV infected persons and 352,371 living AIDS patients [5]. Promoting safe sex to control sexual transmission would greatly contribute to the prevention of HIV transmission, especially the correct and continuous use of condoms in the sexually active population, as condom use is one of the most effective strategies in preventing the spread of HIV in both the general and high-risk populations [6].

Fisher [7] was the first to describe the relationship between HIV prevention information, motivation, and behavioral skills as an information-motivation-behavioral skill (IMB) model, which can easily be transformed into an intervention practice [8, 9]. Many interventions to prevent HIV are based on this IMB model. For example, the IMB model significantly predicts condom use in male contacts, female sex workers, patients with sexually transmitted infections (STI), and students [10, 11]. Through the intervention of using an IMB model structural design, Ybarra et al. [12] and Sun et al. [13] found that condom use increased in MSM, contacts, and students.

The development of ICT provides a potential solution to the people at risk for HIV or living with HIV disease. Social media platforms, such as Twitter, are new sources of HIV/AIDS-related knowledge, attitudes, and practices. Chat-based education programs, web-based treatment educational systems, and online search information could be used to prevent HIV/AIDS [14]. An increasing number of people are using social media, and research shows that data from ICT can be used for public health monitoring, including identifying HIV-related risk behaviors [14, 15]. ICT, including cell phone interventions, could be a fundamental part of successful communication with people at risk for HIV or living with HIV disease. Xiaofang Zhu et al. [16] and other researchers who have done randomized, controlled trials of mobile phone health intervention in promoting HIV self-detection among Chinese male contacts proved its feasibility, acceptability, and preliminary effects in promoting the use of an oral HIV self-testing kit in Chinese men who have sex with men (MSM). Guo et al. [17, 18] believes that WeChat could be a mode of information transmission for Chinese HIV-infected people to promote drug compliance, mental health, and quality of life.

At present, we know of no studies investigating the integration of ICT-based HIV prevention education into the prediction of condom use for the general population in China. This study is the first to attempt an evaluation of the effects of ICT in the HIV prevention for the general population. Using a convenient sampling method, we conducted a large sample follow-up study in Guangxi, Guangdong, Zhejiang, Jiangxi, and 12 other provinces in China. At the beginning of the study, a baseline survey was conducted, including the general population's view on HIV-related information, motivation, and behaviors (IMB). Subsequently, the ICT intervention (through the WeChat platform) was carried out to collect IMB and other information after a six-month intervention. The IMB model was used to evaluate the effects of ICT on HIV prevention for the general population. We attempted to first to establish an IMB model to evaluate the impact of ICT on the general population, to explore the influencing factors of condom use, to evaluate the preventative effects of ICT intervention in HIV services, and to put forward targeted prevention and control measures in China.

## 2. Methods

This is a national follow-up study on the evaluation of ICT on HIV health education and its effects before and after large sample intervention.

### 2.1. Participants

A total of 997 adults were recruited from 16 provinces in China. In December 2018, the research group recruited 50 volunteers through the university and conducted a two-week training course for the questionnaire survey. The 50 volunteers then returned to their hometowns in January 2019 to recruit participants and conduct a baseline questionnaire survey. The inclusion criteria were (a) aged 18 years or older, (b) normal comprehension ability, (c) ability to use WeChat and receipt information, and (d) willingness to take part in the study. The exclusion criteria for participants recruited were (a) inability to understand and complete the questionnaire and (b) inability to response to ICT intervention or follow-up investigation.

### 2.2. Measurement

Questionnaires were used to survey HIV-related information, motivation, and behavior skills of all participants before and after ICT-based HIV health education. The design of this questionnaire referred to relevant literature [19, 20]. The questionnaire included (1) basic personal information (10 items), including age, family income, and marital status. (2) HIV/AIDS-related knowledge information (18 items) and one 1 point was given for each correct answer and 0 points for each incorrect answer. Questions included “Can a person infected with HIV be seen from his appearance?” “Do ordinary mosquito bites spread HIV?” “Will eating with HIV-infected patients lead to HIV infection?” (3) HIV/AIDS-related motivation (8 items), and the answer for the questions can be answered on 5 different levels and are scored on a scale between 0-4 points. The questions and statements were “Should HIV/AIDS patients be isolated?” “HIV/AIDS patients should be banned from work.” “Children infected with HIV cannot go to school with uninfected children.” “If a salesman is infected with HIV, I am not willing to buy food from him.” “I feel uncomfortable shaking hands with HIV/AIDS patients or infected people.” “People living with HIV/AIDS should not eat at the same table as those who are not at risk.” “What is your partner's attitude toward condoms?” “Are you willing to be a volunteer to care for and help HIV-infected people or patients?” (4) HIV/AIDS-related behavior skills (4 items) can be answered on 3 different levels, scored on a scale between 0–2 points, respectively. The topics were “Do you know how to use condoms?” “Do you persuade your partner to use condoms?” “Do you accept of condoms?” and “How easy is it for you to obtain condoms?” In addition, condom use of all participants was investigated. The answers for condom use ranged from 1 to 4 points based on the frequency of condom use, ranging from “Never” (1 point), “Sometimes” (2 points), “Often” (3 points), to “Always” (4 points).

Alpha (*α*) coefficients were used to test for the reliability of the questionnaire, by SPSS 22.0 software. It is a low reliability when the *α* coefficient is less than 0.6. The reliability value was 0.592 when all questions were included. After a modification according to the parameters, the reliability value was 0.873. Therefore, the modified questionnaire is reliable. The modified questionnaire was used in this study according to the reliability test.

### 2.3. Intervention

After administering the baseline questionnaire survey, ICT was used to carry out a six-month HIV prevention education program for the general population on a WeChat platform. The 997 participants received HIV-related information once a week sent by an official WeChat account named *HIV/AIDS Prevention Tips*. The released HIV-related information including the transmission of HIV, how to perform an HIV test, and how HIV affects human immunity. During this period, the 50 volunteers needed to establish the WeChat group for their respective participants. Each week, all participants were given HIV-related knowledge published by the official WeChat account. Volunteers were given a reminder to read and send screen shots to the WeChat group on time. In October 2019, the research group conducted a second questionnaire to collect the data on HIV-related information, motivation, and behavior skills.

### 2.4. Statistical Analysis

Data from the questionnaires were imported into SPSS 22.0, and statistical analysis was carried out using SPSS 22.0 software. The composition ratio was used to describe the basic characteristics of the study subjects, and a paired *t* test was used to analyze and compare the information, motivation, behavioral skills, and condom use before and after the ICT intervention. A paired *t* test was also used to compare the HIV/AIDS information scores before and after intervention, and paired chi-square testing was used to compare the correlation rate before and after the ICT intervention. All tests were two-sided, and *P* < 0.05 was considered statistically significant. The IMB model was built using Mplus7.0 analysis software. Based on the IMB model, this study explored the impact of this ICT intervention on the HIV prevention information, motivation to use HIV prevention, confidence in ability to use HIV prevention behavioral skills, and their impact on actual condom use in the general population.

## 3. Results

### 3.1. Basic Characteristics of Research Participants

In this study, a total of 997 questionnaires were sent, and 957 effective questionnaires were recovered, a recovery rate of 96.00%. The proportion of men and women in the study was 43.46% and 56.64%, respectively, with 35.11% participants being between 20–30 years old. A total of 51.84% objects were unmarried, 54.55% had a bachelor degree or higher, 38.04% were students, and 29.26% had no income (Table 1).

The IMB scores of the participants after the ICT intervention (WeChat platform) were under the principle of informed consent, and ICT was used to provide intervention to the respondents. After six months of the ICT intervention, the same questionnaire was used again to investigate the respondents' progress. The scores for information, motivation, and behavior skills (IMB) as well as the frequency of condom use were analyzed by paired *t*-testing. The results in Table 2 that the scores for information, motivation, and behavior skills and the frequency of condom use improved significantly (*P* < 0.001).

### 3.2. AIDS Information Scores before and after the ICT Intervention

As shown in Table 3 and Table 4, the average score of basic information was 5.44 ± 1.92, and the score of information on transmission routes was 6.44 ± 1.11 in the baseline survey, indicating that the information for this part was better understood, while the score for nontransmission routes was the lowest at only 3.80 ± 1.32. After the ICT intervention, the average score of basic information was 7.20 ± 1.78, the score of transmission information was 6.79 ± 0.62, and the score of nontransmission information was 4.56 ± 0.85. Using a paired *t*-test to compare the two groups, there were statistically significant differences in the scores of basic information, transmission information, and nontransmission information (*P* < 0.001). The total average score of HIV/AIDS-related information before intervention was 15.68 ± 3.28 points, and the accuracy rate (average score/total score) was 74.76%. After intervention, the score was 18.54 ± 2.48 points, and the accuracy rate was 88.29%. The difference in scores before and after intervention was statistically significant (*t* = −21.663, *P* < 0.001).

### 3.3. Motivation towards HIV/AIDS before and after the ICT Intervention

The attitude of the participants towards HIV/AIDS patients or infected persons before and after the ICT intervention was compared using a paired chi-square test. The difference was statistically significant for all questions (*P* < 0.001, Table 5).

### 3.4. Behavior Skills of HIV/AIDS Prevention before and after the ICT Intervention

After the ICT intervention, the behavioral skills of the HIV/AIDS prevention were compared with a paired chi-square test. The differences were statistically significant for all questions (*P* < 0.001, Table 6).

### 3.5. The IMB Model

According to the results of the questionnaire in which 1 point was given for a correct answer and 0 points was given for an incorrect answer, the final score was be taken as an indicator of HIV/AIDS-related information. The mean score of all subjects was 17.08 ± 3.25. In terms of motivation, there were 6 quantitative phenotypic questions, scored on a scale between 0–4 points, (e.g., “Patients with HIV/AIDS should not be allowed to work” and “Children with HIV cannot go to school with uninfected children”). Behavior skills related to the HIV/AIDS prevention were made up of 4 quantitative phenotypic questions (e.g., “Do you know how to use condoms?” “Do you persuade your partner to use condoms?” “Your acceptance of condoms?” and “How easy is it for you to get condoms?”).

The structural equation model was established by using Mplus7.0 analysis software. The fitting index and regression parameters of the model were then analyzed RMSEA = 0.060 < 0.08, CFI = 0.952 > 0.3, and TLI = 0.942 > 0.9. The model had strong structural validity and fit.

**Figure 1:**
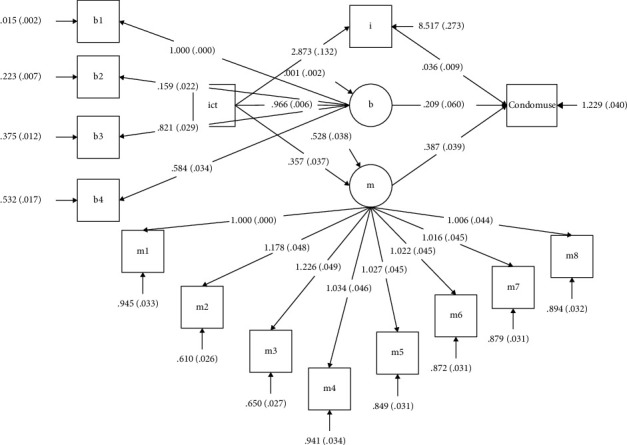
IMB model of ICT interventions for HIV prevention and condom use.

ICT use had a significant regressive effect on motivation (*β* = 0.237, *P* < 0.001), behavior skills (*β* = 0.997, *P* < 0.001), and information (*β* = 0.441, *P* < 0.001), while motivation (*β* = 0.196, *P* < 0.001), behavior skills (*β* = 0.207, *P* < 0.001), and information (*β* = 0.092, *P* < 0.001) had a significant regressive effect on condom use. As shown in Figure 1, receiving ICT intervention services can not only promote HIV/AIDS prevention information, motivation, and behavior skills but can also indirectly promote the use of condoms.

## 4. Discussion

The results of this study show that the baseline survey (administered before the intervention) found that the score of HIV/AIDS nontransmission routes was the lowest, which may be because most HIV health education ignored the knowledge of nontransmission routes, which poses difficulty for the prevention of HIV and leads to misunderstandings and excessive avoidance of those infected with HIV. After 6 months of health education on the HIV prevention by ICT (WeChat platform), the score of HIV/AIDS-related knowledge improved significantly. The awareness rate of HIV/AIDS-related knowledge is 97.81%, and the accuracy rate of basic knowledge, transmission knowledge, and nontransmission knowledge is about 90%. At the same time, the survey found that the improvement of basic knowledge of HIV/AIDS was the most significant after the ICT intervention. It shows that intervention through ICT can function to promote HIV health education. Therefore, it is necessary to carry out all-around and multiangle health education for the general population, especially related to education of HIV/AIDS knowledge of nontransmission routes which is useful for the elimination of points of confusion about prevention knowledge for the general population, so as to reduce panic in the general population regarding HIV and to improve prevention behaviors of HIV infection in the general population.

We found that participants' attitude towards HIV/AIDS had improved significantly after the ICT intervention. However, Wang et al. [21] found that the use of WeChat by college students did not have any significant impact on their attitudes. This is mainly because, firstly, Wang et al.'s intervention time was only 4 weeks, whereas our intervention lasted for 6 months. Furthermore, our volunteers were responsible for urging participants to read the WeChat information on time and ensure the effective implementation of the ICT HIV/AIDS intervention. Secondly, college students have a high level of HIV/AIDS-related information awareness, while the general population in this study has a relatively low level of AIDS-related information awareness. Our study also showed videos concerning skills and precautions of condom use on the ICT platform so that the general population can learn proper condom-use skills effectively. Therefore, after 6 months of intervention using the ICT platform, HIV/AIDS prevention behavioral skills of the research subjects have improved significantly, and most of them can spread and practice newly acquired knowledge by persuading their partners to use condoms, so that HIV/AIDS prevention information learned from the ICT platform has been consolidated.

Fisher's IMB model indicated that information, motivation, and behavior skills were the fundamental factors of HIV/AIDS prevention behaviors, and a person must possess adequate information, motivation, and the necessary behavior skills to learn HIV prevention behaviors [22]. This study used ICT to provide intervention to 997 participants on the WeChat platform. Through the IMB model, the research found that information had the least impact on condom use (*β* = 0.092, *P* < 0.001), followed by motivation (*β* = 0.196, *P* < 0.001), with behavioral skills having the most impact (*β* = 0.207, *P* < 0.001). These results are consistent with the research results of Fisher and Yetao Luo [22–24], which showed that behavior skills were a very important factor in adopting preventative behaviors. In the future, intervention on HIV prevention in the general population should focus on the HIV/AIDS behavior skills, such as teaching participants how to use condoms correctly, improving their acceptance of condoms, and enhancing communication with partners on the use of condoms, etc.

The advantages of the ICT-based intervention include its stability, low cost, and its ability to popularize interventions in general [14]. Prevention and intervention plans are most effective when they are familiar, easy to use, and individually customized [25]. Therefore, people are increasingly inclined to use ICT to implement HIV prevention and intervention. Zhu et al. [16] and Guo et al. [17, 18] proved the feasibility, acceptability, and preliminary effectiveness of ICT health education. In this study, after the ICT intervention in the prevention and treatment of HIV infection and AIDS in the general population, we found that behavioral skills were the most affected, followed by knowledge, while motivation of the general population was the least affected.

Using ICT to carry out health education related to HIV/AIDS is easy to use and easy to promote. When designing educational ICT health content, focusing on the intervention content of behavior skills can directly change AIDS prevention behaviors of the general population, so as to reduce the risk of the spread of HIV in the general population.

In conclusion, ICT intervention services can not only promote the improvement of HIV/AIDS prevention information, motivation, and behavior skills but also promote the use of condoms. Findings from this study can be used to inform the development of a convenient, low cost, easily disseminated tool to help prevent HIV infection among general population.

## Figures and Tables

**Table 1:** Basic characteristics of research participants.

	*N*	%
Gender		
Male	415	43.46
Female	542	56.64
Age (years)		
≤19	208	21.73
20-30	336	35.11
31-40	174	18.18
41-50	183	19.12
51-59	56	5.85
Marital status		
Unmarried	496	51.84
Married	441	46.08
Cohabitation	10	1.04
Divorce or bereavement	10	1.04
Education		
Illiteracy	9	0.94
Primary school	45	4.70
Middle school	89	9.30
High school	137	14.30
Junior college	155	16.20
Bachelor's degree or above	522	54.55
Occupation		
Public institution personnel/civil servants	136	14.21
Enterprise personnel/workers	87	9.10
Clerk	106	11.08
Self-employed	56	5.85
Freelance	78	8.15
Farmer	57	5.96
Unemployed	8	0.84
Students	364	38.04
Other	65	6.79
Source of income		
None	134	14.00
Children	7	0.73
Parents	222	23.20
Part-time job	122	12.75
Regular wages	308	32.18
Business	83	8.67
Farmer	36	3.76
Other	45	4.70
Income (for a month)		
No income	280	29.26
Below 1000 yuan ($143)	60	6.27
1001-2000 yuan ($143-$286)	95	9.93
2001-3000 yuan ($287-$431)	110	11.49
3001-4000 yuan ($432-$574)	114	11.91
4001-5000 yuan ($575-$718)	94	9.82
5001 yuan ($719)	204	21.32
Total	957	957 (100.00)

**Table 2:** The IMB scores of the participants after 6 months intervention.

Constructs	Group	*N*	^−^ *χ* ± *S*	*t*	*P*
I (information)	BeforeICT intervention	957	15.68 ± 3.28	-21.663	<0.001
	AfterICT intervention		18.54 ± 2.48		
M (motivation)	BeforeICT intervention	957	14.47 ± 4.26	-8.334	<0.001
	AfterICT intervention		16.06 ± 4.11		
B (behavior skills)	BeforeICT intervention	957	2.26 ± 1.08	-51.141	<0.001
	AfterICT intervention		4.74 ± 1.04		
Condom use	BeforeICT intervention	957	2.19 ± 1.15	-8.422	<0.001
	AfterICT intervention		2.64 ± 1.15		

**Table 3:** HIV/AIDS information scores before and after the ICT intervention ($\bar{x}\pm \text{S}$).

Constructs	Before ICT		After ICT		t	*P*
	*N*	$\bar{x}\pm \text{S}$	*N*	$\bar{x}\pm \text{S}$		
Basic information	957	5.44 ± 1.92	957	7.20 ± 1.78	-20.933	<0.001
Information of transmission route	957	6.44 ± 1.11	957	6.79 ± 0.62	-8.324	<0.001
Information of nontransmission route	957	3.80 ± 1.32	957	4.56 ± 0.85	-15.161	<0.001
Total score of HIV/AIDS-related information	957	15.70 ± 3.28	957	18.54 ± 2.48	-21.66	<0.001

**Table 4:** Nontransmission routes of HIV/AIDS information correct rates before and after the ICT intervention.

Question	Before ICT (%)	After ICT (%)	*χ* ^2^	*P*
1. Can mosquito bites spread HIV?	702 (85.89)	864 (90.28)	92.172	<0.001
2. Can eating with HIV/AIDS patients?	857 (89.55)	929 (97.07)	43.403	<0.001
3. Does common contact (shaking hands or hugging) with HIV/AIDS patients infect with HIV?	895 (93.52)	933 (97.49)	17.581	<0.001
4. Can the use of public toilets infect with HIV?	509 (53.19)	807 (84.33)	215.982	<0.001
Total	957 (100.00)	957 (100.00)		

**Table 5:** Motivation towards HIV/AIDS before and after ICT (*N*, %).

Question	Motivation	Before ICT (%)	After ICT (%)	*χ* ^2^	*P*
HIV/AIDS patients should be isolated	Agree	224 (13.41)	184 (19.23)	17.794	<0.001
	Neutral	330 (34.48)	278 (29.05)		
	Disagree	403 (42.11)	495 (51.72)		
HIV/AIDS patients should not be allowed to work	Agree	103 (10.76)	59 (6.17)	32.472	<0.001
	Neutral	291 (30.41)	220 (22.99)		
	Disagree	563 (58.83)	678 (70.85)		
Children infected with HIV cannot go to school with uninfected children	Agree	212 (22.15)	130 (13.58)	47.764	<0.001
	Neutral	287 (29.99)	223 (23.30)		
	Disagree	458 (47.86)	604 (63.11)		
If a salesman infected with HIV, I do not want to buy food from him	Agree	234 (24.45)	146 (15.26)	29.157	<0.001
	Neutral	296 (30.93)	293 (30.62)		
	Disagree	427 (44.62)	518 (54.13)		
I feel uncomfortable if I shake hands with someone infected with HIV	Agree	199 (20.79)	122 (12.75)	42.624	<0.001
	Neutral	312 (32.60)	253 (26.44)		
	Disagree	446 (46.60)	582 (60.82)		
People infected with HIV should not eat at the same table as those who are not ill	Agree	171 (17.87)	98 (10.24)	38.944	<0.001
	Neutral	294 (30.72)	242 (25.29)		
	Disagree	492 (51.41)	617 (64.47)		
What is your partner's attitude toward condoms?	Agree	536 (56.00)	417 (43.57)	31.726	<0.001
	Neutral	186 (19.44)	213 (22.26)		
	Disagree	235 (24.56)	327 (34.17)		
Are you willing to be a volunteer to care for and help HIV-infected people or patients?	Agree	517 (54.02)	428 (43.72)	19.109	<0.001
	Neutral	191 (19.96)	207 (21.63)		
	Disagree	249 (26.02)	322 (33.65)		
Total		957 (100.00)	957 (100.00)		

**Table 6:** Behavior skills of the HIV/AIDS prevention before and after the ICT intervention (*N*, %).

Questions	Behavior	Before ICT (%)	After ICT (%)	*χ* ^2^	*P*
Do you know how to use condoms?	Yes	17 (1.78)	942 (98.43)	1788.148	<0.001
	No/not sure	940 (98.22)	15 (1.57)		
Do you persuade your partner to use condoms?	Yes	271 (28.32)	413 (43.16)	45.873	<0.001
	No/not sure	686 (71.68)	544 (56.84)		
Your acceptance of condoms	Yes	367 (38.35)	868 (90.70)	597.309	<0.001
	No/not sure	323 (33.75)	85 (8.88)		
	Yes	267 (27.90)	4 (0.42)		
How easy is it for you to get condoms?	No/not sure	233 (24.35)	242 (25.29)	855.827	<0.001
	Yes	630 (65.83)	71 (7.42)		
	No/not sure	94 (9.82)	644 (67.29)		
Total		957 (100.00)	957 (100.00)		

## Data Availability

The datasets generated and/or analyzed during the current study are not publicly available due to ethical and legal reasons but are available from the corresponding author on reasonable request.

## References

[1] Howitt P., Darzi A., Yang G. Z. (2012). Technologies for global health. *Lancet*.

[2] Lewis T., Synowiec C., Lagomarsino G., Schweitzer J. (2012). E-health in low- and middle-income countries: findings from the center for health market innovations. *Bulletin of the World Health Organization*.

[3] Gour N., Srivastava D. (2010). Knowledge of computer among healthcare professionals of India: a key toward e-health. *Telemedicine journal and e-health : the official journal of the American Telemedicine Association*.

[4] Ward J. P. T., Gordon J., Field M. J., Lehmann H. P. (2001). Communication and information technology in medical education. *Lancet*.

[5] Zolton J. R., Lindner P. G., Terry N., DeCherney A. H., Hill M. J. (2020). Gonadotropins versus oral ovarian stimulation agents for unexplained infertility: a systematic review and meta-analysis. *Fertility and Sterility*.

[6] Moreno R., Nababan H. Y., Ota E. (2014). Structural and community-level interventions for increasing condom use to prevent the transmission of HIV and other sexually transmitted infections. *The Cochrane Database of Systematic Reviews*.

[7] Fisher C. M. (2012). Adapting the information-motivation-behavioral skills model: predicting HIV-related sexual risk among sexual minority youth. *Health education & behavior : the official publication of the Society for Public Health Education*.

[8] Fisher J. D., Fisher W. A. (1992). Changing AIDS-risk behavior. *Psychological Bulletin*.

[9] Fisher J. D., Fisher W. A., Peterson J. L., DiClemente R. J. (2000). Theoretical approaches to individual-level change in HIV risk behavior. *Handbook of HIV Prevention*.

[10] Kalichman S. C., Picciano J. F., Roffman R. A. (2008). Motivation to reduce HIV risk behaviors in the context of the information, motivation and behavioral skills (IMB) model of HIV prevention. *Journal of Health Psychology*.

[11] Zhang H., Liao M., Nie X. (2011). Predictors of consistent condom use based on the information-motivation-behavioral skills (IMB) model among female sex workers in Jinan, China. *BMC Public Health*.

[12] Ybarra M. L., Liu W., Prescott T. L., Phillips G., Mustanski B. (2018). The effect of a text messaging based HIV prevention program on sexual minority male youths: a national evaluation of information, motivation and behavioral skills in a randomized controlled trial of Guy2Guy. *AIDS and Behavior*.

[13] Sun W. H., Wong C. K. H., Wong W. C. W. (2017). A peer-led, social media-delivered, safer sex intervention for Chinese college students: randomized controlled trial. *Journal of Medical Internet Research*.

[14] Young S. D., Rivers C., Lewis B. (2014). Methods of using real-time social media technologies for detection and remote monitoring of HIV outcomes. *Preventive Medicine*.

[15] Finlayson A. E., Greaves F., Ali F. R. (2012). Technologies for global health. *Lancet*.

[16] Zhu X., Zhang W., Operario D. (2019). Effects of a mobile health intervention to promote HIV self-testing with MSM in China: a randomized controlled trial. *AIDS and Behavior*.

[17] Guo Y., Hong Y. A., Qiao J. (2018). Run4Love, a mHealth (WeChat-based) intervention to improve mental health of people living with HIV: a randomized controlled trial protocol. *BMC Public Health*.

[18] Guo Y., Xu Z., Qiao J. (2018). Development and feasibility testing of an mHealth (text message and WeChat) intervention to improve the medication adherence and quality of life of people living with HIV in China: pilot randomized controlled trial. *JMIR mHealth and uHealth*.

[19] Wang Y., Jia M., Yuan D. (2019). Assessing consistent condom use among migrant men who have sex with men in Shanghai, China: validation of an information-motivation-behavioural skills model. *BMC Infectious Diseases*.

[20] Jiang H., Chen X., Li J., Tan Z., Cheng W., Yang Y. (2019). Predictors of condom use behavior among men who have sex with men in China using a modified information-motivation-behavioral skills (IMB) model. *BMC Public Health*.

[21] Wang Y., Bie W., Wang X. (2018). Application of Wechat healtheducation in AIDS prevention among university students. *Chinese Evidence-Based Nursing*.

[22] Fisher J. D., Fisher W. A., Misovich S. J., Kimble D. L., Malloy T. E. (1996). Changing AIDS risk behavior: effects of an intervention emphasizing AIDS risk reduction information, motivation, and behavioral skills in a college student population. *Health Psychology : Official Journal of the Division of Health Psychology, American Psychological Association*.

[23] Shell D. F., Newman I. M., Perry C. M., Folsom A. R. (2011). Changing intentions to use smokeless tobacco: an application of the IMB model. *American Journal of Health Behavior*.

[24] Luo Y., Jing D., Ye M. (2018). Preventive behaviors of migrating MSM against HIV/AIDS based on the information-motivation-behavioral skills model. *Chinese Journal of Preventive Medicine*.

[25] Labrique A. B., Vasudevan L., Kochi E., Fabricant R., Mehl G. (2013). mHealth innovations as health system strengthening tools: 12 common applications and a visual framework. *Global Health: Science and Practice*.

